# Chorea and retinal vessel occlusion in a patient with systemic lupus erythematosus

**Published:** 2013

**Authors:** Vahid-Reza Ostovan, Askar Ghorbani

**Affiliations:** Department of Neurology, Shariati Hospital, Tehran University of Medical Sciences AND Iranian Center of Neurological Research, Tehran, Iran

**Keywords:** Chorea, Retinal Vessel Occlusion, Systemic Lupus Erythematosus, Antiphospholipid Syndrome

## Abstract

Various neurological complications occur in primary or secondary antiphospholipid syndrome (APS) consisting of cerebrovascular attacks, ocular events, dementia, seizure, chorea, and transverse myelopathy that are all related to the titer of antiphospholipid antibodies (aPL). We report a patient with chorea and retinal vessel occlusion as manifestations of systemic lupus erythematosus (SLE) and APS. A 27-year-old man presented with progressive visual field defect and decreases visual acuity of right eye (OD) as well as involuntary movements in both hands and slurred speech. Investigations led to the diagnosis of SLE and APS. Anticoagulant and immunosuppressant was started for him. As his condition improved, the prednisolone was gradually tapered. This is the first case report of concomitant retinal vessel occlusion and chorea in SLE and APS.

## Introduction

Antiphospholipid syndrome (APS) is characterized by recurrent thrombotic events, abortion, and thrombocytopenia. It is associated with the production of antiphospholipid antibodies (aPL), including anticardiolipin (aCL), lupus anticoagulant (LA), and anti-beta 2-glycoprotein I, which present on two or more measurements taken 3 months apart.^1^

On the basis of the absence or presence of another autoimmune disease, the syndrome classified as primary or secondary, respectively.^2^ Various neurological complications occur in primary or secondary APS consisting of cerebrovascular attacks, ocular events, dementia, seizure, chorea, and transverse myelopathy that are related to the titer of aPL.^3^ Herein, we report an unusual case of APS and systemic lupus erythematosus (SLE) that presented with chorea and central retinal artery and vein occlusion.

## Case Report

A 27-year-old man presented with 2 weeks history of progressive visual field defect and decreased visual acuity of right eye. He also had complaints of involuntary movements in both hands and slurred speech. He had history of similar abnormal movements in hands 5 years ago that had improved spontaneously and rheumatologic work-up were negative. He denied any history of malar rash, aphthous ulcer and thrombotic event.

**Figure 1:**
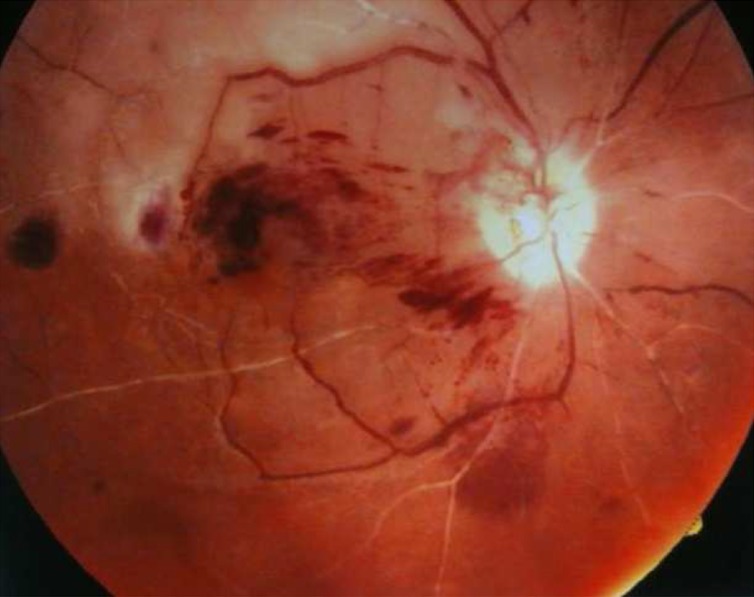
Fundus photograph of right eye indicating central retinal vein and central retinal artery occlusion

Physical examination demonstrated choreic movements in his hands. Visual acuity of right eye (OD) was finger count in 1-meter distance and left eye (OS) was 20/20. In addition, he had papillary afferent defect on right side. Fundoscopic examination revealed a paled and edematous retina, engorged and tortuous retinal veins, flame-shaped hemorrhages and swollen optic disc (Figure 1).

He had slurred speech. Other examination was unremarkable. Laboratory investigations showed thrombocytopenia (platelet = 80000/microliter), and 4500 mg/day proteinuria in 24-hour urine.

According to history, physical examination, and paraclinical findings the rheumatologic work-up was done for him.

**Figure 2:**
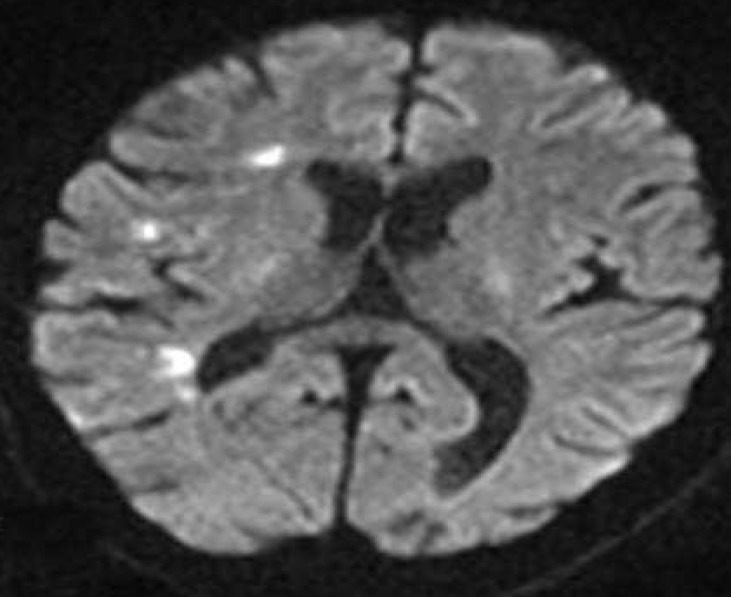
Diffusion weighted magnetic resonance imaging revealed multiple new lacunar infarcts in subcortical white matter of right cerebral hemisphere

The level of antinuclear antibody was 1/20, anti-double-stranded DNA was 60 IU/ml (normal up to 25 IU/ml), anticardiolipin IgG > 120 U/ml (normal up to 10 U/ml), antiphospholipid IgG > 100 U/ml (normal up to 10 U/ml) and lupus anticoagulant was positive. Brain magnetic resonance imaging (MRI) showed old lacunar infarct in the head of the right caudate and multiple new infarctions in right hemisphere (Figure 2).

Ophthalmologic examination revealed optic atrophy, macular hemorrhage and extensive vascular necrosis. Anticoagulant and immunosuppressant was started for him. On the follow up, 3 months after treatment, visual acuity of right eye did not change, but choreic movements improved and proteinuria resolved.

## Discussion

APS diagnostic criteria include a positive test of aCL antibody or LA antibody, measured twice or more with a interval of 3 months and one of clinical presentation of APS such as arterial and/or venous thrombosis or thrombocytopenia;^1^ therefore, diagnosis of APS was made for the reported case.

According to American College of Rheumatology classification criteria, diagnosis of SLE can be established.^4^ Neurological manifestations in APS consist of thrombotic events, psychiatric features and a range of non-thrombotic syndrome.^3^

Several studies suggest the association between the level of aCL and risk of thrombosis; thus the moderate-to-high titer of aCL antibody correlates with an increased risk of thrombosis,^3^ similar to our presented case that had high titer of aCL. We proposed that the ocular symptoms in our patient were due to vascular thrombosis secondary to APS rather than vasculitis by SLE.

Firstly, SLE vasculitis tends to be symmetrically bilateral,^5^ unlike the present case which had unilateral involvement. Secondly, classic SLE retinopathy manifests itself by cotton wool spot^6^ that was not seen in our case. Ophthalmologic manifestations include anterior ischemic optic neuropathy, preretinal hemorrhage, amaurosis fugax; however, the characteristic feature of APS is retinal vessels occlusion which is more common in individuals with high titer of aCL.^7^

Chorea is a rare, reversible neurological manifestations of SLE and is strongly associated with the presence of aPL.^3^ Two-thirds of patients with chorea and APS had SLE and 96% of cases were female with mean age of 23 years. The chorea was bilateral in 55% and imaging studies showed cerebral infarction in one-third of cases.^3^

Asherson et al.^8^ hypothesized that the pathogenic mechanism of chorea can be related to striatal binding of aPL. This mechanism can describe the presence of chorea in the absence of imaging abnormality and responsiveness to immunosuppressive medications other than anti dopaminergics. Gutrecht et al.^9^ reported an unusual case of primary APS with combination of chorea and retinal arterial thrombosis without any evidence of SLE.

This is the first reported case of simultaneous occurrence of chorea and retinal vessels occlusion as the initial manifestation of secondary APS. Baizabal-Carvello et al.^10^ revealed that aPL at the onset of chorea may be negative and later on, IgM and IgG became positive. As a result, it is crucial to recheck aPL 6 months later if there is clinically suspicious of APS. This case report also showed high titer of aCL despite negative result in the initial presentation of chorea 5 years ago.

## Conclusion

It is necessary to consider APS in patients with unexplained retinal vessels occlusion and/or chorea and/or cerebral infarction especially in young. Even negative result of aPL at initial presentation does not rule out the diagnosis. It is important to promptly diagnose and treat APS and other connective tissue disorders because of high morbidity and mortality of them.
